# Supplementary material for: SPOP Deregulation Improves the Radiation Response of Prostate Cancer Models by Impairing DNA Damage Repair
Source: Cancers (Basel). 2020 Jun 4;12(6):1462. doi: 10.3390/cancers12061462 (PMC7352729; doi:10.3390/cancers12061462)
Supplement: Supplementary file 1 [file cancers-12-01462-s001.zip › cancers-783382-suppl.-proofs/Supplementary Table 1.pdf]

Supplementary Table 1. Plating efficiency values of DU145 and PC-3 cells transfected with WT or mutant SPOP

	Plating Efficiency *	
Cells	**DU145**	**PC-3**
**Empty** vector	0.196 ± 0.002 **	0.253 ± 0.013
**WT** SPOP	0.196 ± 0.004	0.246 ± 0.018
**Y87N** SPOP	0.176 ± 0.009	0.179 ± 0.005
**K129E** SPOP	0.175 ± 0.004	0.179 ± 0.004
**F133V** SPOP	0.172 ± 0.001	0.178 ± 0.016

* N° of colonies/N° of plated cells
** Mean value ± SD from 3 independent experiments
