# Supplementary figures and images for: SPOP Deregulation Improves the Radiation Response of Prostate Cancer Models by Impairing DNA Damage Repair
Source: Cancers (Basel). 2020 Jun 4;12(6):1462. doi: 10.3390/cancers12061462 (PMC7352729; doi:10.3390/cancers12061462)

Figure 3C

---

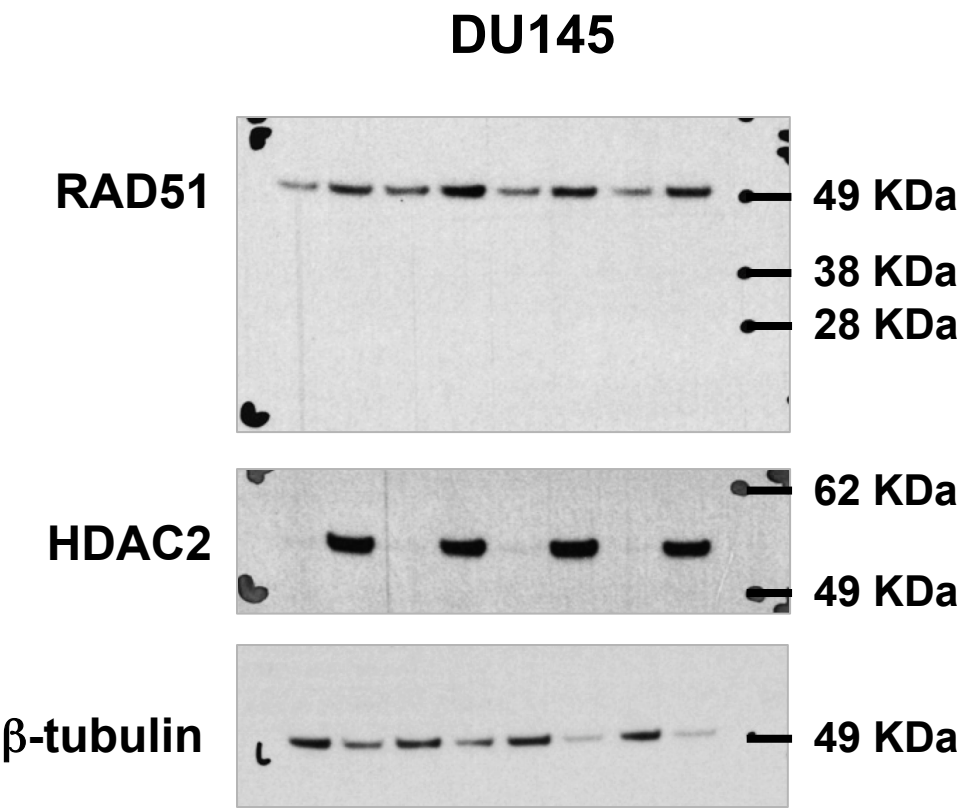

**Figure 4B**

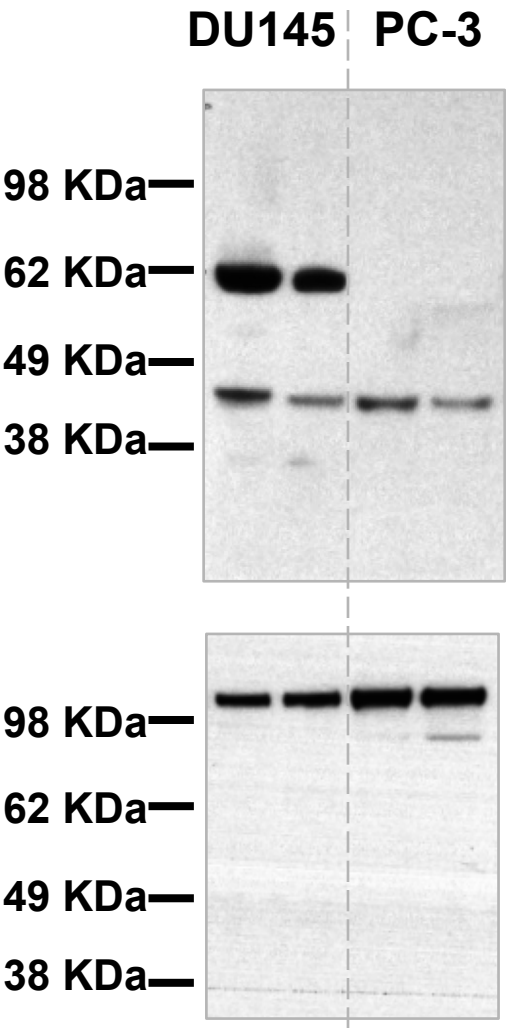

← **SPOP** →

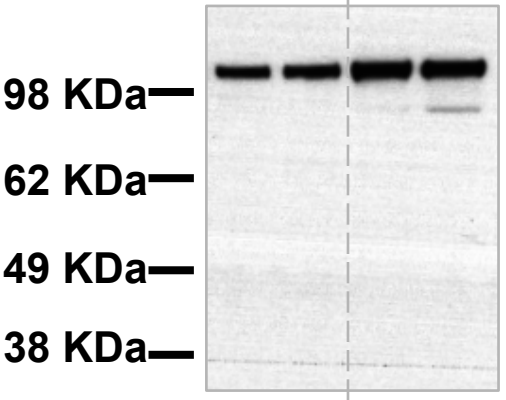

← **Vinculin** →

**Figure 4F**

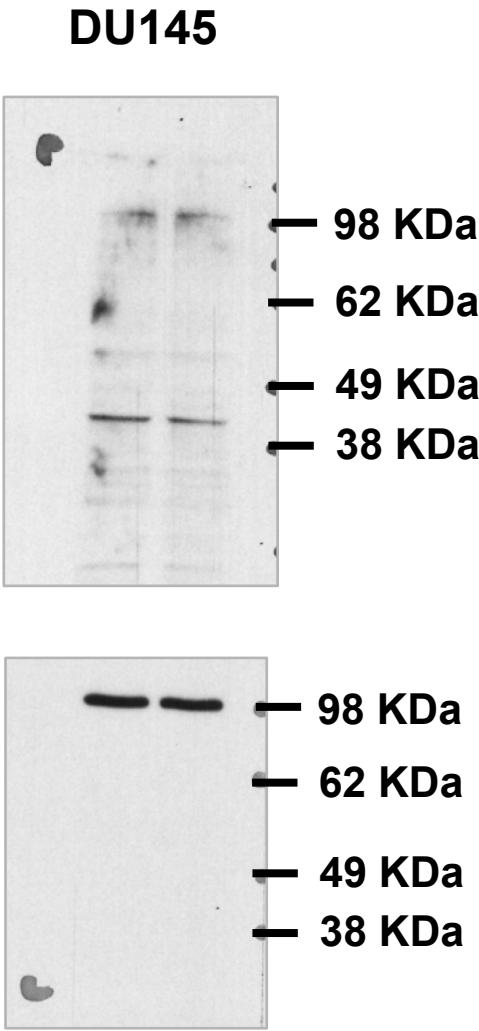

**Figure 5B**

---

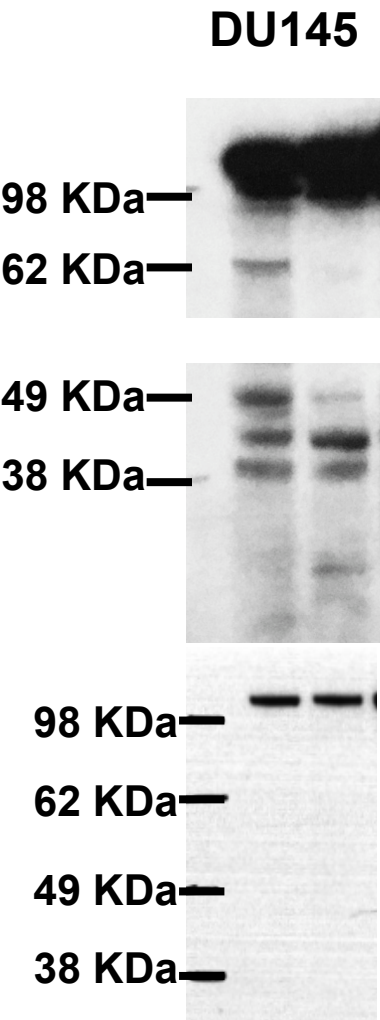

**Figure 5B**

---

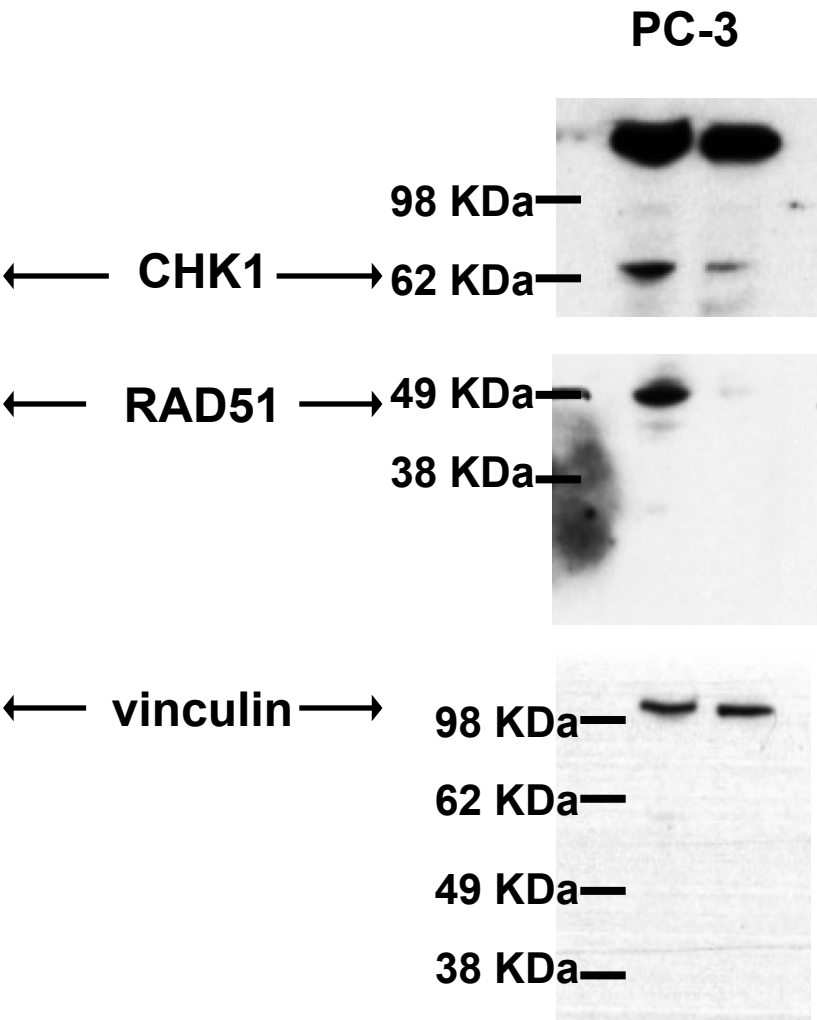

Supplement: Supplementary file 1 [file cancers-12-01462-s001.zip › cancers-783382-suppl.-proofs/Supplementary_Figure S1.pdf]
